# A New Type of Proton Coordination in an F_1_F_o_-ATP Synthase Rotor Ring

**DOI:** 10.1371/journal.pbio.1000443

**Published:** 2010-08-03

**Authors:** Laura Preiss, Özkan Yildiz, David B. Hicks, Terry A. Krulwich, Thomas Meier

**Affiliations:** 1 Department of Structural Biology, Max-Planck Institute of Biophysics, Frankfurt, Germany; 2 Department of Pharmacology and Systems Therapeutics, Mount Sinai School of Medicine, New York, New York, United States of America; 3 Cluster of Excellence Macromolecular Complexes, Max-Planck Institute of Biophysics, Frankfurt, Germany; University of California Berkeley, United States of America

## Abstract

We solved the crystal structure of a novel type of c-ring isolated from *Bacillus pseudofirmus* OF4 at 2.5 Å, revealing a cylinder with a tridecameric stoichiometry, a central pore, and an overall shape that is distinct from those reported thus far. Within the groove of two neighboring c-subunits, the conserved glutamate of the outer helix shares the proton with a bound water molecule which itself is coordinated by three other amino acids of outer helices. Although none of the inner helices contributes to ion binding and the glutamate has no other hydrogen bonding partner than the water oxygen, the site remains in a stable, *ion-locked* conformation that represents the functional state present at the c-ring/membrane interface during rotation. This structure reveals a new, third type of ion coordination in ATP synthases. It appears in the ion binding site of an alkaliphile in which it represents a finely tuned adaptation of the proton affinity during the reaction cycle.

## Abstract 2

The high-resolution structure of the rotor ring from alkaliphilic *Bacillus pseudofirmus* OF4 reveals a new type of ion binding in F_1_F_o_-ATP synthases.

## Author Summary

Like the wind turbines that generate electricity, the F_1_F_o_-ATP synthases are natural “ion turbines” each made up of a stator and a rotor that turns, when driven by a flow of ions, to generate the cell's energy supply of ATP. The F_o_ motor rotates by reversible binding and release of coupling ions that flow down the electrochemical ion gradient across the cytoplasmic cell membrane (in the case of bacteria) or intracellular organelle membranes (in the case of eukaryotic cells). Here, we present the structure of a rotor (c-)ring from a *Bacillus* species (*B. pseudofirmus* OF4) determined at high-resolution by X-ray crystallography. This bacterium prefers alkaline environments where the concentration of protons (H^+^) is lower outside than inside the cell – the inverse of the situation usually found in organisms that prefer neutral or acidic environments. The amino acid sequence of the protein subunits in this rotor, nevertheless, has features common to an important group of ATP synthases in organisms from bacteria to man. The structure reveals a new type of ion binding in which a protonated glutamate residue in the protein associates with a water molecule. This finding raises the possibility considered by Nobel laureate Paul Boyer several decades ago that a hydronium ion (a protonated water molecule, H_3_O^+^), rather than a proton alone, might be the coupling species that energizes ATP synthesis. Also, it demonstrates the finely tuned adaptation of ATP synthase rotor rings and their ion-binding sites to the specific requirements of different organisms.

## Introduction

Most living cells depend upon the adenosine triphosphate (ATP) generated by F_1_F_o_-ATP synthases that are energized by a proton- or a sodium-motive force (pmf, smf). These multi-subunit enzymes contain a cytoplasmic F_1_ catalytic domain (subunits α_3_β_3_γδε) that is connected with a membrane-embedded F_o_ domain (ab_2_c_10–15_ in bacteria) by a central (γε) and peripheral (b_2_δ) stalk. Energetically down-hill ion translocation across the membrane through the F_o_ complex is mediated by successive interactions between the stator a-subunit and a rotor ring (c-ring). Translocation involves ion binding to an unoccupied c-subunit, rotation, and subsequent ion release. The c-ring is attached to the γε stalk subunits so that c-ring rotation causes rotation of the stalk. The inherently elastic [1] and asymmetric γ-subunit extends into the α_3_β_3_ headpiece [2] and by rotation [3] induces conformational changes [4] in the catalytic β-subunits, which results in ATP synthesis.

**Figure 1:**
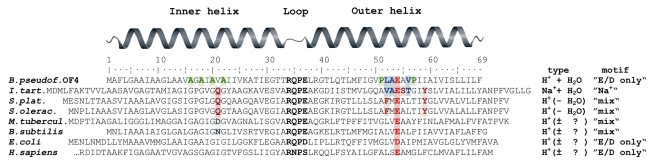
Amino acid sequence alignment of c-subunits. The c-subunits of selected species were aligned according to their cytoplasmic loop region (bold). Residues structurally proven to be involved in ion coordination shown in this work and in [7] are shown in dark red (Na^+^ or H^+^) and blue (H_2_O+H^+^). The conserved ion-binding glutamate/aspartate is highlighted in red for all species. The motifs (AxAxAxA and PxxExxP) [14] in *Bacillus pseudofirmus* OF4 (numbering) are highlighted in green. The type of ion binding in the respective ATP synthase is indicated on the right side, together with their corresponding binding motifs. Among these types: E/D-only is defined in the text, “Na^+^” is a sodium-coupled rotor with a complete Na^+^-binding motif [7], and “mix” is a proton-coupled rotor that has elements of the Na^+^ motif (highlighted in grey), e.g. a polar (or charged) residue at positions required for Na^+^ coordination [7],[33].

In the Na^+^-binding c_11_ ring from *Ilyobacter tartaricus* [5] and the H^+^-binding c_15_ ring from *Spirulina platensis* [6], the translocated ions are bound within the groove of two adjacent c-subunits in a coordination network including a conserved carboxylate (Glu). In both cases, the ion is further coordinated by a precise network of residues, several of which are common to both organisms (Figure 1). The ion specificity of these two systems is determined by several factors including the geometry and distances of the ion coordination network, and a water molecule [7] providing a coordination site for Na^+^. The ion binding specificity of ATP synthases in various cells is adapted to the physiological requirements of the organism, and the different binding motifs observed presumably reflect these adaptations. The range of the ion-binding motif includes variations from complete Na^+^-binding signatures to c-subunits where the conserved carboxylate (E/D) of the C-terminal helix is the only residue that can be predicted with confidence to play a role in ion coordination (e.g., in *Escherichia coli* or *Homo sapiens*, Figure 1). On this basis, we here assign the name “E/D-only” to the c-subunits of this sub-class of proton-coupled ATP synthases.

Alkaliphilic *Bacillus* species are among the bacteria having proton-coupled ATP synthases [8] with E/D-only c-subunits (Figure 1). The extreme alkaliphile *Bacillus pseudofirmus* OF4 grows by oxidative phosphorylation with cytoplasmic pH values maintained 1.5–2.3 pH units below the high external pH (up to 11) of the medium [9]. The existence of this reversed ΔpH poses a major thermodynamic problem, with which these cells must cope. Among a variety of adaptive strategies to resolve the energetic problem [10], some special adaptations of the ATP synthase itself have evolved: latent ATPase activity [9],[11], a-subunit modification [12],[13], and in particular, specific adaptations of the c-subunit sequence [14] resulting in a large c-ring width with more c-subunits [15]. The adaptations to alkaliphilic conditions in an ATP synthase rotor, with a widely found but structurally uncharacterized E/D-only motif, made its c-ring an attractive candidate for an X-ray diffraction study.

## Results

### Structure of the *Bacillus pseudofirmus* OF4 c_13_ Ring

**Figure 2:**
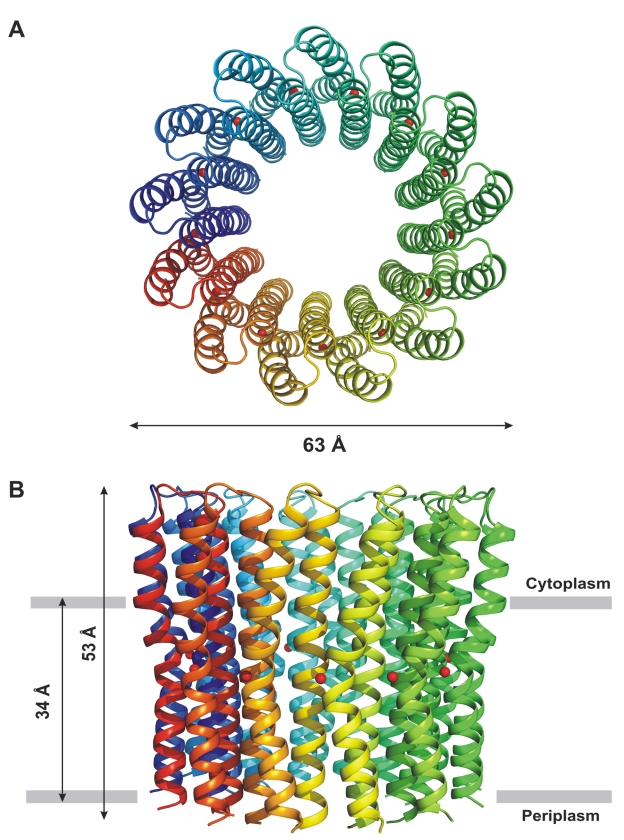
Structure of the c_13_ ring of the alkaliphilic *Bacillus pseudofirmus* OF4 ATP synthase. The c-subunits are shown in different colors in ribbon representation. The protonated water molecules are shown as red spheres. (A) View perpendicular to the membrane from the cytoplasm. (B) Side view of the c_13_ ring. The membrane border is indicated with grey bars.

**Figure 3:**
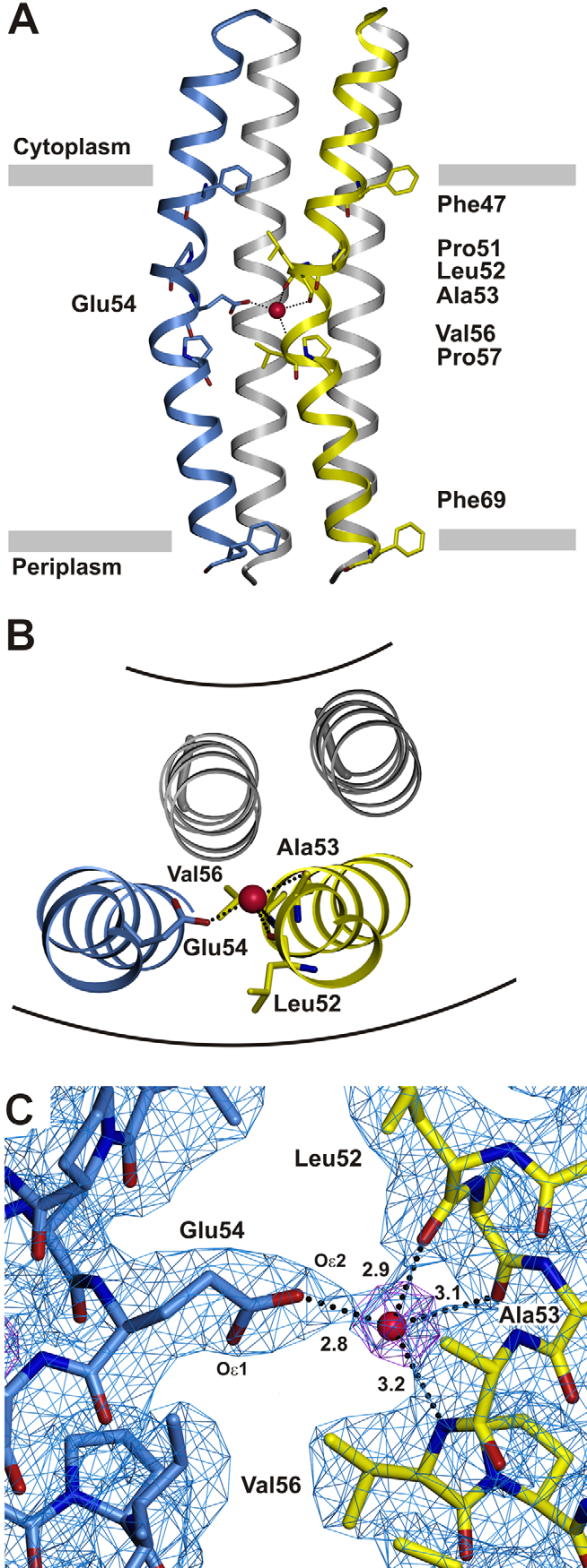
The ion binding site. The c-subunits are shown in different colors as ribbons and the protonated water is shown as a red sphere. Coordination of the ion is indicated by dashed lines. (A) A c_2_-dimer consisting of two adjacent C-terminal α-helices (blue and yellow) and two straighter N-terminal helices (gray). (B) View perpendicular to the membrane focused on the binding side. (C) The binding site at pH 4.5, view from the membrane plane. The electron density (2F_obs_-F_calc_, shown at 1.3 σ) is shown as a blue mesh. The coordinating residues Glu54, Leu52, Ala53, and Val56 are indicated. The omit map for the position of the water oxygen (O) is shown as purple mesh (σ  = 4.5). Distances are in Å.

Three-dimensional crystals of the c-ring from native *Bacillus pseudofirmus* OF4 F_1_F_o_-ATP synthase were obtained and diffracted to 2.5 Å (Table 1). An asymmetric unit contains one complete c-ring, forming crystal contacts with three neighboring, laterally translated c-rings including two loop-to-loop contacts and one loop-to-C-terminus contact. One c-ring is formed by 13 identical c-subunits with a central pore (Figure 2A). Because of the unusually short N- and C-termini typical of *Bacillus* species (Figure 1), it does not extend into the periplasmic space but instead ends at the periplasmic membrane surface, whereas on the cytoplasmic side, the protrusion out of the membrane is comparable to that observed in other c-rings (Figure S1). In contrast with the distinctly hour-glass shape of the c_11_, K_10_, and c_15_ rotor rings [5],[6],[16], this c_13_ ring resembles a “tulip beer glass” appearance, in which the only slightly curved periplasmic side transitions at about the depth of the ion-binding site into a more significantly flared cytoplasmic side (Figure 2B). The c_13_ ring has an outer diameter of 63 Å on the cytoplasmic side, 54 Å on the periplasmic side, and 52 Å in the middle. Each c-subunit consists of two α-helices that are connected by a short cytoplasmic loop (RQPE, residues 34 to 37). The N-terminal α-helices (residues 1 to 33) form an inner ring, surrounded by an outer ring of the C-terminal α-helices (residues 38 to 69) in staggered position. While the inner helices are remarkably straight (Figure 3A) and only slightly tilted by ∼5° toward the c-ring axis (as seen from the periplasm), the outer helices are more curved and have a kink in the middle of the helix at the key carboxylate residue (Glu54). Above that kink, toward the cytoplasmic loop, the outer helices are bent ∼30° to the membrane vertical and form a convex surface shape on the outside of the ring. Toward the periplasmic side the helices remain more straight but still tilt ∼10° against the membrane vertical (Figure 3). The outer surface of the c_13_ ring (Figure S2A) is encircled by a large hydrophobic region forming membrane borders at the level of Phe47 on the cytoplasmic side and Phe69 at the very end of the ring on the periplasmic side with a height of 34 Å. This region is the contact region of the c_13_ ring with the hydrophobic part of a membrane, in good agreement with previously described membrane borders for other c- (or K-) rings [5],[6],[16]. Except for the cytoplasmic end, the surface in the inner pore of the c_13_ ring is overall hydrophobic and binds detergent molecules (Figure S2B), which apparently replace phospholipids. A second leaflet of phospholipids/detergent molecules at the periplasmic side of the c-ring could only be discerned partially from the electron densities, but its existence can be inferred. In support of this notion are data showing a plug of phospholipids [17] in the *I. tartaricus* c_11_ ring and also atomic force microscopy topographs from *Bacillus* sp. strain TA2.A1 c_13_ ring [15].

**Table 1:** Data collection and refinement statistics.

	c_13_ Ring
*Data collection*	
Space group	P2_1_
Cell dimensions	
*a*, *b*, *c* (Å)	74.18, 97.34, 121.24
* α*, *β*, *γ* (°)	90.0, 104.7, 90.0
Resolution (Å)	20–2.5 (2.6–2.5)*
*R*_meas_	9.8 (98.5)
*R_mergd-F_*	20.4 (119.8)
*I*/σ*_I_*	11.7 (1.6)
Completeness (%)	98.8 (92.8)
Redundancy	3.64 (3.1)
*Refinement*	
Resolution (Å)	20–2.5
Number of reflections	57,052
*R*_work_/*R*_free_	18.76%/23.45%
Number of atoms	6,753
Protein	6,370
Ligand/ion	309
Water	84
*B*-factors	55.2
Protein	52.46
Ligand/ion	111.9
Water	54.7
R.m.s. deviations	
Bond lengths (Å)	0.007
Bond angles (°)	0.932

*Values in parentheses are for highest resolution shell. The data are strong, with only little anisotropy, to 2.6 Å resolution. Inclusion of data to 2.5 Å resolution, despite high R-merge values, helped improve the electron density at the ion binding sites.

Special amino acid sequence motifs are found in the c-subunits of alkaliphilic *Bacillus* species (Figure 1). An altered glycine motif in the inner helices is shown to affect the structure and biochemistry of c-rings [14],[15]. The 63 Å diameter of the c_13_ ring width (Figure 2A) is larger than expected if compared with the diameters of the c_11_ and c_15_ c-rings [5],[6], 50 Å and 65 Å, respectively, since it is closer to the diameter of the c_15_ ring. The more relaxed positioning of the helices in the alkaliphile ring is caused by the structural impact of the alanine motif AxAxAxA that replaces the glycine motif GxGxGxG most often found in the first helix coding region of c-subunits. Similarly, replacement of two glycines with serines also accounts for the larger than anticipated c-ring diameter observed in the ATP synthase of *Bacillus* TA2.A1 [15]. On the outer helices of the c_13_ ring from an alkaliphile, two prolines located one helix turn below and above the ion-binding glutamate can be identified in a motif (PxxExxP) [14] in which the first proline, Pro51, is specific for alkaliphiles [13]. Both prolines break the regular α-helix hydrogen bonding pattern and cause helix bends; these two motifs of this c-ring are important factors that have an impact on the c_13_ diameter and ion binding as well as the overall “tulip beer glass” shape of the complex (Figure S1).

### The Ion Binding Site

Ion binding in all c-rings includes a conserved outer helix Glu (or Asp). In the c_13_ ring of *B. pseudofirmus* OF4, this residue (Glu54) is located ∼6 Å above the middle of the membrane (Figure 3) toward the cytoplasmic side. At the pH (4.5) used in the cryo-protection buffer, Glu54 is protonated. Two outer helices from neighboring c-subunits form an ion binding site (Figure 3C). During structure refinement a sphere-shaped density in 2F_obs_-F_calc_ as well as in the omit map remained unassigned. In close proximity of this density center (2.8-3.2 Å), four atoms were identified. One of these belongs to the side chain carboxyl oxygen (Oε2) of Glu54, whereas the three others originate from the backbone carbonyls of Leu52 and Ala53 and from the backbone nitrogen of Val56. The observed distances and the arrangement of the four associated hydrogen donor/acceptor sites around this density are in almost pyramidal arrangement and the hydrogen atom positions lie on a plane. Such an arrangement resembles internal protonated water molecules (hydronium ion) [18] in other proteins.

**Figure 4:**
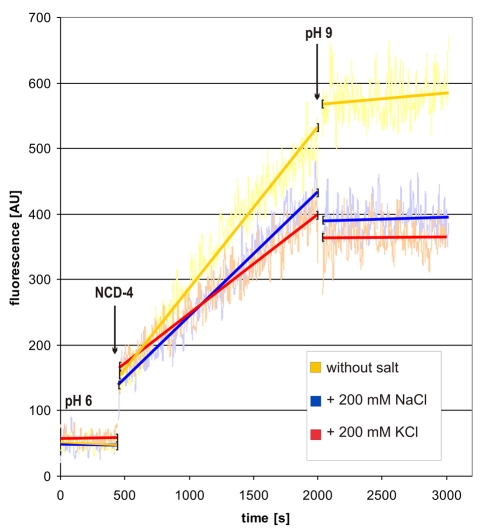
Kinetics of the modification of the ion-coordinating Glu54 of detergent-solubilized c_13_ ring from *B. pseudofirmus* OF4 with NCD-4 in response to pH, NaCl, and KCl. The continuous fluorescence of a sample containing *B. pseudofirmus* c_13_ ring was recorded (yellow). The reaction of NCD-4 with Glu54 was started by the addition of 100 µM NCD-4 and the increase of fluorescence at λ = 465 nm was monitored. An increase of pH to 9.0 immediately and significantly decelerates the reaction. The presence of 200 mM NaCl (blue) had no notable effect beyond effects also made by the presence of 200 mM KCl (red) on the reaction kinetics.

Electron densities of certain cations (e.g., Na^+^) or oxygen atoms from water molecules are similar and difficult to distinguish by X-ray crystallography at the given resolution. However, several lines of evidence indicate the density seen in the binding pocket of this c_13_ ring should be interpreted as water rather than Na^+^. The hydrogen-bonding distance and angle geometry for the ligands are in the typical range for water molecules in proteins, as they are also found, for example, in carboxypeptidase (Figure S3) or in the protonated water cluster of bacteriorhodopsin [19]. In contrast, the mean distances for Na^+^ coordination such as those in the Na^+^-binding c_11_ ring [7] are significantly shorter (∼2.3 Å) [20] than observed in the c_13_ ring. For direct experimental evidence we used NCD-4, a fluorescent analogue of the ATP synthase inhibitor DCCD, which is known to react covalently with protonated glutamates/aspartates in c-subunits [21]. Figure 4 illustrates the time-dependent labeling of detergent-solubilized c_13_ ring at pH 6.0. Consistent with dependence of labeling on protonation of the carboxylate, an increase of pH to 9.0 immediately and significantly decelerates the reaction. This observation has also been reported by others [22],[23] and a control experiment showed that the labeling by NCD-4 continued to increase linearly when the labeling time was extended to 9,000 s without an alkaline pH shift (Figure S4). The marked deceleration upon imposition of an alkaline pH shift also resembles the DCCD labeling pattern of the proton-coupled c_15_ ring as a function of pH (unpublished data). Most importantly, the presence of 200 mM Na^+^ shows no dramatic influence on the labeling kinetics and addition of 200 mM K^+^ leads to comparable effects to Na^+^. Whereas the presence of salts, either Na^+^ or K^+^, presumably causes minor changes on, for example, detergent micelles, water availability, or fluorophore quenching [24], the lack of a major Na^+^-specific effect on NCD-4 binding rates contrasts sharply with the immediate and strong Na^+^-protective effect of a much lower Na^+^ concentration (15 mM) on the Na^+^-binding c_11_ ring [25]. This key evidence for H^+^ rather than Na^+^ binding of the *B. pseudofirmus* OF4 c_13_ ring is fully consistent with biochemical studies on the pmf- (but not smf-) dependent *B. pseudofirmus* OF4 cells [26] and its H^+^-dependent ATP synthase [9]. The result furthermore suggests that the ion binding site is highly selective for H^+^ over Na^+^ essentially under any (physiological) condition with a minimum concentration excess of ∼10^8^ Na^+^ (200 mM) over H^+^ (pH 9.0). Taken together, the findings indicate that the density observed in the binding site of the c_13_ ring of the *B. pseudofirmus* OF4 ATP synthase is an oxygen atom with four valences. The data show that the proton must be located in between the atom positions of Glu54(Oε1/2) and the water oxygen (O).

## Discussion

### Comparison of the c-Rings and Ion Binding Sites

In contrast to other rotor ring structures [5],[6],[16], no residues from the inner helices contribute to ion coordination in the c_13_ ring (Figure 3B and Figure S1B). In the c_11_ and c_15_ rings a proline (Pro28 and Pro25, respectively) is involved in kinking the inner helices (not shown), thereby allowing the hydrogen bonding of the glutamine (Gln32 and Gln29, respectively) with the glutamate on the outer helix. This proline and glutamine are replaced by a glycine and valine, respectively, in the c_13_ ring. Consequently, the inner helices form a complete α-helical hydrogen bonding pattern, retain a straight shape, and cannot hydrogen bond with the ion-binding glutamate.

**Figure 5:**
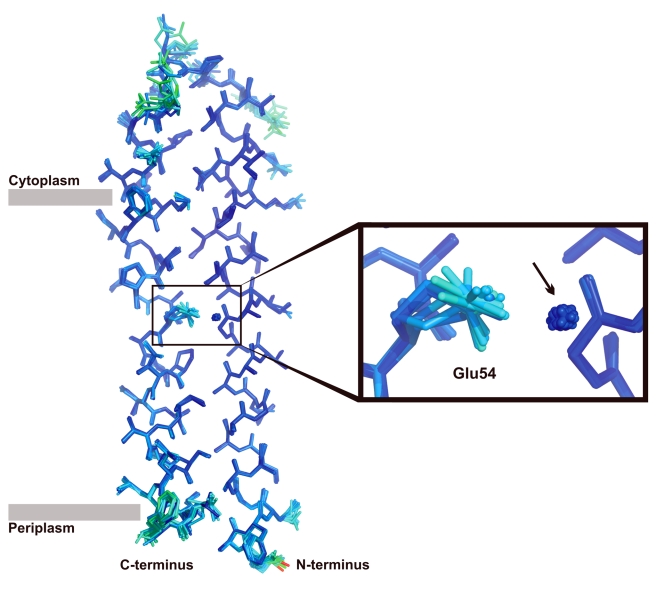
Overlay of 13 c-subunits from *B. pseudofirmus* OF4 c_13_ ring. The color code is given in B-factor (temperature factor) from low (blue) to high (red). The zoomed region shows the ion binding site with the rotameric states of Glu54 as discussed in the text. The position of the water is indicated by an arrow. The position of the membrane border at the outer helix is indicated with grey bars.

A second notable difference of the ion binding site in the c_13_ ring as compared with the c_11_ and c_15_ rings is visible at the second oxygen of the glutamate (Glu54 Oε1). Whereas in c_11_ and c_15_ this oxygen forms a hydrogen bond with a tyrosine from the adjacent outer helix, such an interaction is missing in the c_13_ ring (Figure 3C and Figure S1B). The hydrogen bonding network of Glu54 in the c_13_ ring is therefore reduced to one bond only compared to the others. The additional freedom allows more rotameric flexibility of the glutamate carboxylate. This property becomes impressively visible in an overlay of 13 single c-subunits taken from one asymmetric unit (Figure 5). Notably, although the carboxyl group appears in different rotameric states in the crystal structure, the distance of the closest Glu54 oxygen to the water oxygen remains in bonding distance (2.6–3.1 Å) across all binding sites of the c_13_ ring.

**Figure 6:**
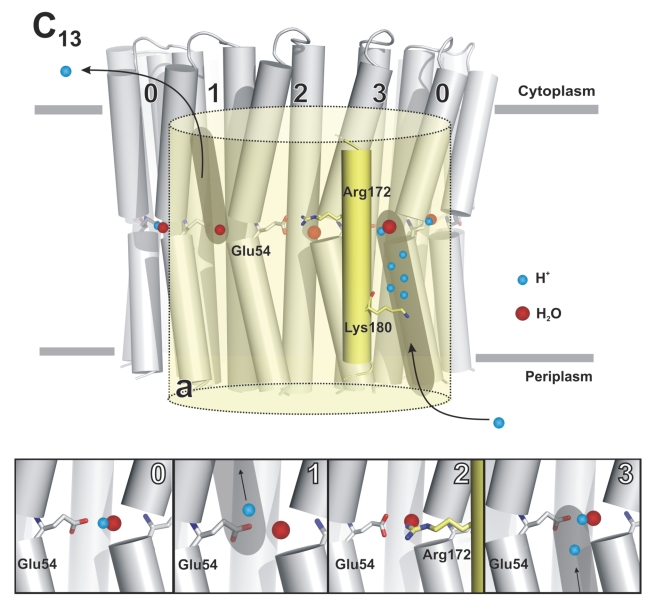
Ion translocation model for the F_1_F_o_-ATP synthase of *Bacillus pseudofirmus* OF4. The model shows the c_13_ ring (rotor, grey) with the neighboring a-subunit (stator, yellow) and the view is slanted to the membrane plane. A selection of c-subunits are shown with the ion coordinating Glu54 and helix 4 of the a-subunit [43] with Arg172 and Lys180 [13]. In ATP synthesis mode of the enzyme, the rotor moves from left to right. Two access pathways to and from the binding sites in the membrane [44],[45] are indicated in grey. The grey bars mark the membrane border. The four stages (0, 1, 2, and 3) of the ion translocation mechanism are indicated in a close-up view and further described in Text S1.

The subtle but important differences in the H-bonding network geometry allow a fine-tuning of the p*K*_a_ of the carboxylic acid [27] and serve to optimize the required solvation energy [28] which is necessary to unlock the site and allow ion release and reloading during the ion translocation mechanism in the F_o_ complex (Figure 6 and Text S1). Fine tuning of these parameters is of crucial importance within the a/c-ring interface, where the rotor binding sites pass a more hydrophilic environment [29] (and J. D. Faraldo-Gómez, personal communication with TM) that is somewhat unique because of the adaptations in both the a- and c-subunits of the alkaliphile [13],[14],[15]. By contrast, while the ion-binding site is in contact with the hydrophobic barrier of the lipid phase, during the long rotation cycle of the rotor ring, the glutamate is expected to be neutralized. The structural data suggest that under these conditions the ion binding site of the c_13_ ring remains in the *ion-locked* state, much in analogy with the *Na^+^*- and *H^+^-locked* states in the c_11_ and c_15_ ring, respectively [5],[6].

The amino acid residues involved in the coordination of the water are exactly at the same positions of the c_13_ ring as those that coordinate the water in the Na^+^-binding c_11_ ring (Figure 1 and Figure S1B) [7]. This commonality of binding pattern underlines the evolutionary and functionally conserved relationship between the pmf- and smf-driven systems. The smf-driven ATP synthases have been suggested to be evolutionary pioneers in the establishment of the modern ATP synthases [30]. If this hypothesis is correct, the E/D-only type such as that seen in the *B. pseudofirmus* OF4 c_13_ ring and in the mixed type (c_15_ ring) primarily found in light-driven systems (chloroplasts, cyanobacteria) could be derivatives of the c_11_ basic structure from an evolutionary point of view. Rather small differences in the amino acid sequences of the c-subunits apparently account for the different ion binding types, which are phenotypically manifested in the differently coupled and differently environmentally adapted ATP synthases.

### A Hydronium Ion (H_3_O^+^) as Coupling Ion for F_1_F_o_-ATP Synthases?

Hydrogen atoms have a very weak X-ray diffracting power and their electron density often does not match with the exact position of the nucleus. Therefore, from a crystallographic point of view, at current resolution, the data do not allow the distinction between a protonated glutamate associating with a water molecule and a hydronium ion as a separate species. The scenario of a hydronium ion as a possible coupling ion species in F_1_F_o_-ATP synthases was proposed for consideration by Boyer more than 20 years ago [31]. Later, experimental differences in the pH-dependent inhibition kinetics of Na^+^- and H^+^-ATP synthases were interpreted to be in support of this hypothesis [32], but recent high-resolution structure data on the cyanobacterial [6] and chloroplast [33] c-rings clearly conflict with this as a general hypothesis. The possibility raised by the structural data presented here that this E/D-only type of c-ring may ultimately conform to Boyer's suggestion awaits further experimental (and/or quantum mechanical) analyses.

### Conclusions

This work shows a new type of proton coordination in an F_1_F_o_-ATP synthase rotor ring. An additional electron density within the protonated ion binding site corresponds to a water molecule (but not Na^+^). It is evident that the coordination network of the water itself, in analogy with the stable water coordination network in the Na^+^-binding c_11_ ring, is a stabilizing and therefore a structural part of this c-ring. The presence of the water has been shown to enhance the Na^+^-binding affinity in the Na^+^-binding c_11_ ring [7]. Given this observation we propose that the water in the c_13_ ring binding pocket also enhances the proton affinity. High affinity rotor binding sites are of central importance for all ATP synthases but are especially important for ATP synthases of bacteria that grow in alkaline environments [13],[14].

Some of the novel details of this c-ring are likely to be specific to *Bacillus* species growing at high pH [34], especially those differences in shape that relate directly to alkaliphile-specific motifs. Perhaps the novel manner in which a water participates in proton binding is also a consequence of adaptation of the ATP synthase to alkaliphily. Further structural analysis of c-rings from the large groups of non-alkaliphilic species harboring the E/D-only motif is necessary to clarify the precise role of such water molecules in the ion translocation process. It may reveal the presence of this ion binding type throughout a broader subset of H^+^-coupled rotors, where it influences both ion affinity and selectivity during torque generation in the F_o_ motors of the H^+^-dependent F-type ATP synthases, and possibly also for the ion-driven motors known from V-type and A-type ATPases/synthases.

## Materials and Methods

### Purification and Crystallization of the c-Ring from *Bacillus pseudofirmus* OF4

The ATP synthase was purified from *B. pseudofirmus* OF4 in which a six histidine tag was inserted after the N-terminal methionine in the chromosomal gene encoding the β-subunit of the ATP synthase. The complex was extracted from everted vesicles with 1% β-dodecyl maltoside in the presence of 3 mg/ml soybean asolectin and purified by affinity chromatography on NiNTA agarose. The isolation of the c-ring was carried out according to [25]. To improve purity of the sample, the c-ring was concentrated by ultrafiltration with an Amicon tube with a molecular weight cut-off of 10’000 (Millipore GmbH, Schwalbach, Germany), incubated with 1.5% Foscholine-12 (w/v) at 45°C for 10 min, and run on a sucrosegradient [15],[35]. The c-ring containing fractions were concentrated by Hydroxyapatite (BioGelHT, Bio-Rad, Munich, Germany) [36] and dialyzed for 12 h (10 mM Tris/HCl pH 8.0) at 4°C. The c-ring was further concentrated using polyethylene glycol (PEG) [37] to a final concentration of 2.5 mg/ml (bicinchoninic acid assay, Pierce, Rockford, IL, USA). Crystals were grown by vapor diffusion in hanging drops at 18°C to a size of approx. 200×100×100 µm^3^. The c-ring sample was supplied with 1% (w/v) of β-undecyl maltoside and mixed with crystallization buffer (0.1 M sodium acetate, pH 4.3) and 20% PEG 400 (v/v). Before flash-freezing in liquid nitrogen, the rod shaped clear crystals were transferred for 2 min into a buffer containing 30% PEG 400 (v/v), 0.1 M sodium acetate pH 4.5, and 0.05% β-dodecyl maltoside (w/v).

### NCD-4 Labeling Reactions

A 60 µl sample (0.2 mg/ml) of purified c_13_ from *Bacillus pseudofirmus* OF4 in 12.5 mM MES-HCl (pH 6.0) buffer and 0.05% β-dodecyl maltoside (w/v) was used. Continuous increase of fluorescence was recorded with an F-4500 Hitachi Fluorescence Spectrophotometer (λ_ex_ = 342 nm, λ_em_ = 465 nm). The reaction was started by the addition of 0.6 µl of NCD-4 (Invitrogen Inc.) from a 10 mM stock solution in dimethylformamide. After 2,000 s, the rate of reaction was greatly reduced by addition of 11 µl of 1 M Tris/HCl pH 9.0. The time required for the addition of these compounds (NCD-4 and Tris buffer) was approximately 5 s in both cases.

### Data Collection, Structure Determination, and Refinement

Data to 2.5 Å resolution were collected from a single crystal at the Max-Planck beamline X10SA (PX-II) at the Swiss Light Source (SLS, Villigen, Schwitzerland) and processed using the XDS package (Kabsch, 1993). The structure was determined by molecular replacement using PHASER (McCoy, 2007) with two bundles of six subunits from the structure of the c_15_ ring from *Spirulina platensis* [6] as search model. Model bias was removed by density modification and solvent flattening with RESOLVE [38]. Iterative cycles of model building and refinement were performed using COOT [39] and phenix.refine of the PHENIX package [40], respectively. During refinement, no non-crystallographic symmetry operation was applied. The refinement resulted in electron density maps that were unambiguously interpretable and after chain fitting the Ramachandran plot shows no outliers. Figures were generated using Povscript [41], POV-ray (http://www.povray.org), and Pymol [42]. Electrostatic potential distribution was generated using Pymol [42].

#### Data deposition

The atomic coordinates and structure factors of the *Bacillus pseudofirmus* OF4 c_13_ ring have been deposited with accession code 2x2v.

## Supporting Information

Figure S1 **Comparison of c-ring structures from** ***Ilyobacter tartaricus*** **,** ***Bacillus pseudofirmus*** **OF4, and** ***Spirulina platensis.*** (A) The c-subunits are shown in ribbon representation. Side views of the c-rings from *I. tartaricus* (yellow, 1yce and 2wgm), *B. pseudofirmus* OF4 (blue, 2x2v), and *S. platensis* (green, 2wie). The membrane border is indicated with grey bars. (B) View on the three types of ion binding sites in F-type ATP synthases. c_11_, *I. tartaricus*; c_13_, *B. pseudofirmus* OF4; c_15_, *S. platensis*. The hydrogen bonding network is indicated by dashed lines and the ion/water molecules are shown with small spheres.
(2.44 MB TIF)

Figure S2 **Electrostatic potential distribution of the** ***B. pseudofirmus*** **OF4 c_13_ ring surface.** (A) Side view on the surface. (B) Section through the ring, same view as in (A). Detergent molecules (Foscholine-12) attached to the hydrophobic inner surface are displayed in stick representation (yellow) and helices of the c-ring in ribbon representation. Colors: red, negative; blue, positive; white, neutral. The membrane border is indicated with grey bars.
(0.37 MB TIF)

Figure S3 **Comparison of ion coordination in** ***B. pseudofirmus*** **OF4 c_13_ ring and carboxypeptidase**. (A) Ion coordination in the c_13_ ring from *B. pseudofirmus* OF4. (B) Ion coordination in carboxypeptidase A1 (PDB code 3i1u). The water oxygen at the glutamate is shown as a red sphere. Distances are given in Å. In both cases, the water oxygen has four valences for hydrogen, either four (A) or three (B) of them are forming a hydrogen bonding network with the corresponding protein (complex).
(1.67 MB TIF)

Figure S4 **Long-term kinetics of the modification of the ion-coordinating Glu54 of detergent-solubilized c_13_ ring from** ***B. pseudofirmus*** **OF4 with NCD-4 in the absence and presence of NaCl.** The fluorescence of a sample containing *B. pseudofirmus* c_13_ ring was taken every 20 min at pH 6 in the absence (yellow circles) or presence (blue squares) of 200 mM NaCl. The reaction of NCD-4 with Glu54 was started by the addition of 100 µM NCD-4 and the increase of fluorescence at λ = 438 nm was followed for 9,000 s. The arrow marks the time point, at which the rate of NCD-4 labeling was reduced by shifting the pH to 9 in the experiment shown in Figure 4 (see text). For experimental details see Materials and Methods section.
(0.59 MB TIF)

Text S1 **Text S1 contains Figures S1–S4; Discussion of ion translocation through the** ***B. pseudofirmus*** **OF4 F_o_ complex; and References for Text S1.**
(0.07 MB DOC)

## Abbreviations

ATP: adenosine 5'-triphosphate
DCCD: *N*,*N*'-dicyclohexyl-carbodiimide
NCD-4: *N*-cyclohexyl-*N*'-(4-dimethylamino-α-naphthyl)carbodiimide
pmf: proton motive force
smf: sodium motive force
