# Correction: A New Type of Proton Coordination in an F_1_F_o_-ATP Synthase Rotor Ring

**DOI:** 10.1371/annotation/a025ce51-d0f4-41ca-b5b9-89d9c75cd26d

**Published:** 2010-08-20

**Authors:** Laura Preiss, Özkan Yildiz, David B. Hicks, Terry A. Krulwich, Thomas Meier

The images for Figures S2 and S3 were incorrectly switched. The image that appears as Figure S2 should be Figure S3, and the image that appears as Figure S3 should be Figure S2. The figure legends appear in the correct order. Please view the correct Figure S3 here: 
