# Supplementary figures and images for: A New Type of Proton Coordination in an F1Fo-ATP Synthase Rotor Ring
Source: PLoS Biol. 2010 Aug 3;8(8):e1000443. doi: 10.1371/journal.pbio.1000443 (PMC2914638; doi:10.1371/journal.pbio.1000443)

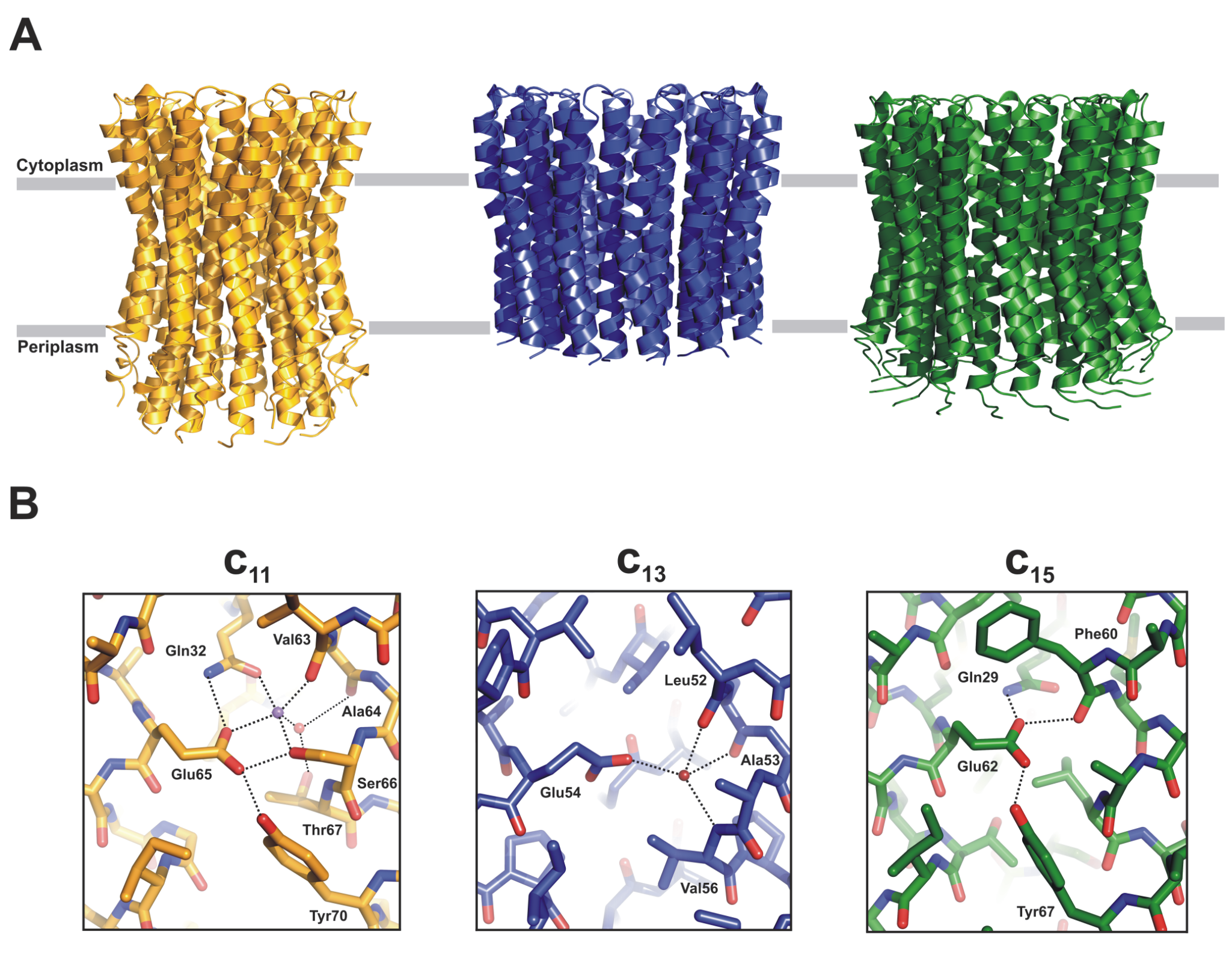

Supplement: Figure S1 — Comparison of c-ring structures from Ilyobacter tartaricus , Bacillus pseudofirmus OF4, and Spirulina platensis. (A) The c-subunits are shown in ribbon representation. Side views of the c-rings from I. tartaricus (yellow, 1yce and 2wgm), B. pseudofirmus OF4 (blue, 2x2v), and S. platensis (green, 2wie). The membrane border is indicated with grey bars. (B) View on the three types of ion binding sites in F-type ATP synthases. c11, I. tartaricus; c13, B. pseudofirmus OF4; c15, S. platensis. The hydrogen bonding network is indicated by dashed lines and the ion/water molecules are shown with small spheres. (2.44 MB TIF) [file pbio.1000443.s001.tif]

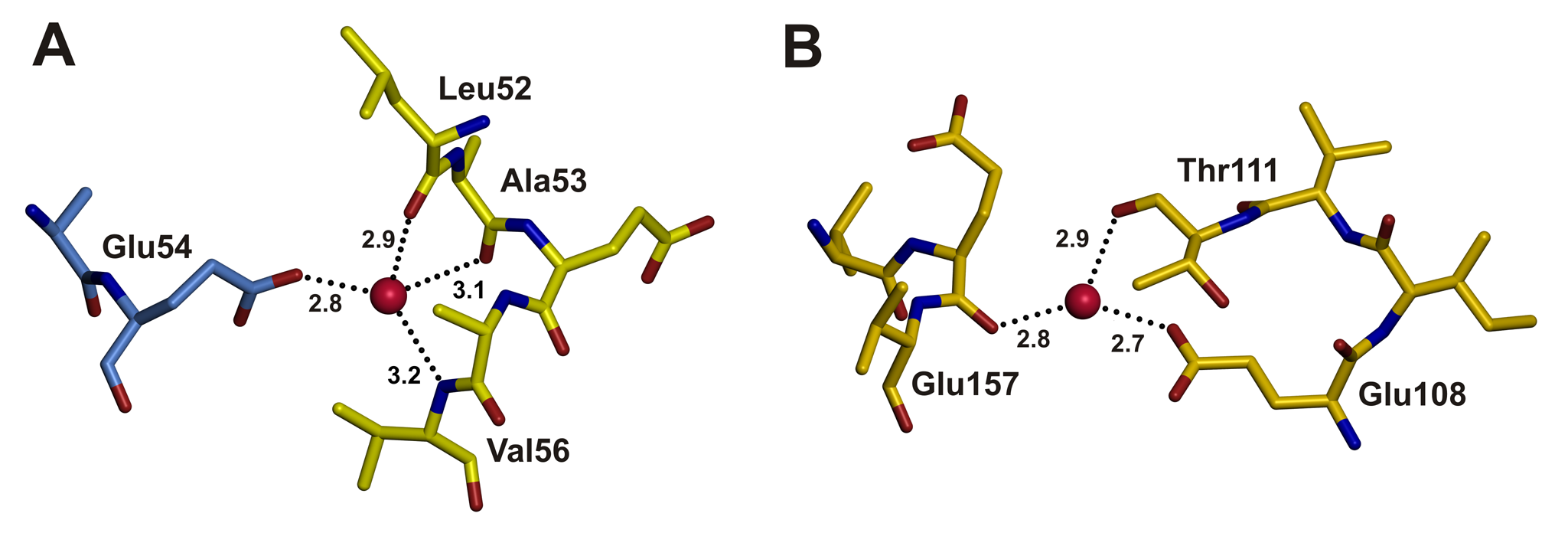

Supplement: Figure S2 — Electrostatic potential distribution of the B. pseudofirmus OF4 c13 ring surface. (A) Side view on the surface. (B) Section through the ring, same view as in (A). Detergent molecules (Foscholine-12) attached to the hydrophobic inner surface are displayed in stick representation (yellow) and helices of the c-ring in ribbon representation. Colors: red, negative; blue, positive; white, neutral. The membrane border is indicated with grey bars. (0.37 MB TIF) [file pbio.1000443.s002.tif]

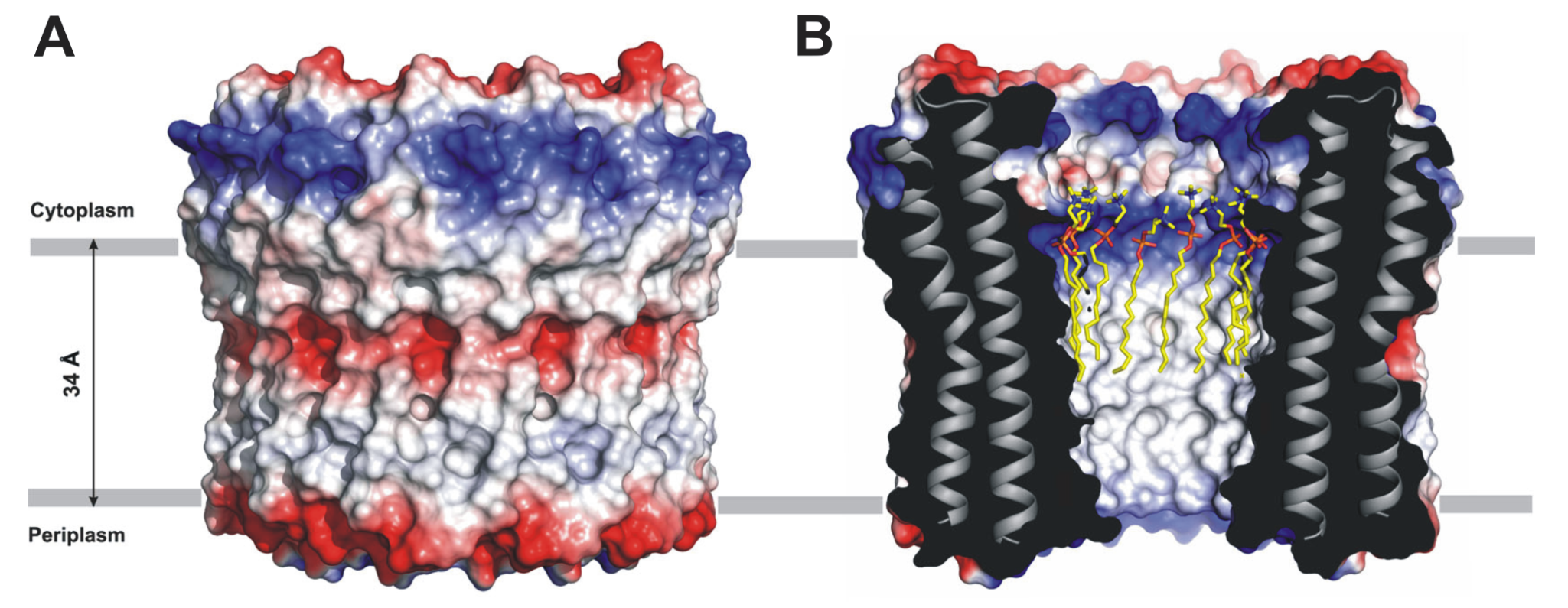

Supplement: Figure S3 — Comparison of ion coordination in B. pseudofirmus OF4 c13 ring and carboxypeptidase. (A) Ion coordination in the c13 ring from B. pseudofirmus OF4. (B) Ion coordination in carboxypeptidase A1 (PDB code 3i1u). The water oxygen at the glutamate is shown as a red sphere. Distances are given in Å. In both cases, the water oxygen has four valences for hydrogen, either four (A) or three (B) of them are forming a hydrogen bonding network with the corresponding protein (complex). (1.67 MB TIF) [file pbio.1000443.s003.tif]

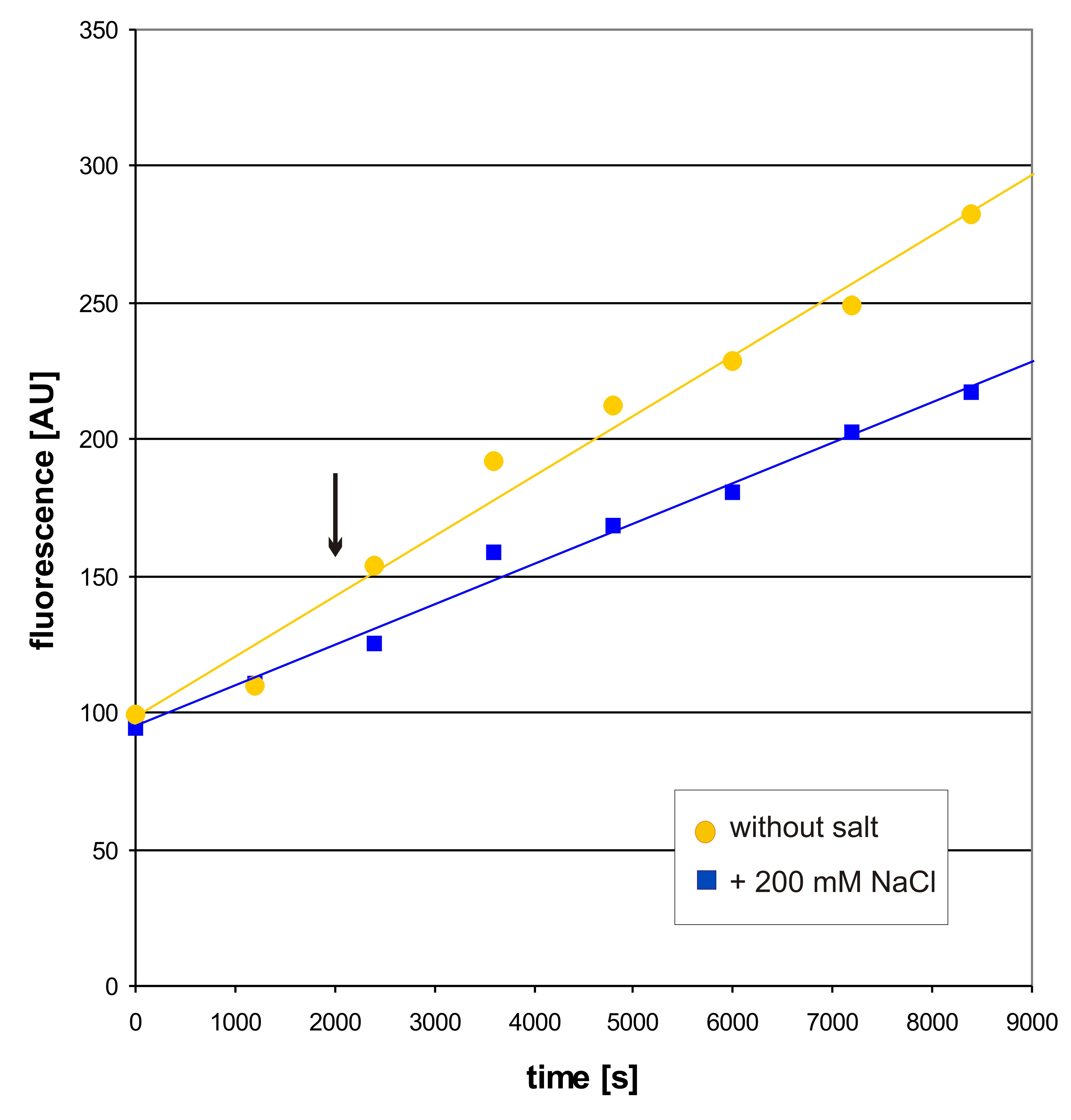

Supplement: Figure S4 — Long-term kinetics of the modification of the ion-coordinating Glu54 of detergent-solubilized c13 ring from B. pseudofirmus OF4 with NCD-4 in the absence and presence of NaCl. The fluorescence of a sample containing B. pseudofirmus c13 ring was taken every 20 min at pH 6 in the absence (yellow circles) or presence (blue squares) of 200 mM NaCl. The reaction of NCD-4 with Glu54 was started by the addition of 100 µM NCD-4 and the increase of fluorescence at λ = 438 nm was followed for 9,000 s. The arrow marks the time point, at which the rate of NCD-4 labeling was reduced by shifting the pH to 9 in the experiment shown in Figure 4 (see text). For experimental details see Materials and Methods section. (0.59 MB TIF) [file pbio.1000443.s004.tif]
