# Supplementary material for: A New Type of Proton Coordination in an F1Fo-ATP Synthase Rotor Ring
Source: PLoS Biol. 2010 Aug 3;8(8):e1000443. doi: 10.1371/journal.pbio.1000443 (PMC2914638; doi:10.1371/journal.pbio.1000443)
Supplement: Text S1 — contains Figures S1–S4; Discussion of ion translocation through the B. pseudofirmus OF4 Fo complex; and References for Text S1. (0.07 MB DOC) [file pbio.1000443.s005.doc]

**TEXT S1 FOR:**

**A new type of proton coordination**

**in an F_1_F_o_-ATP synthase rotor ring**

**Laura Preiss*^1^*, Özkan Yildiz*^1^*, David B. Hicks*^2^*,**

**Terry A. Krulwich*^2^* and Thomas Meier*^1,3,*^***

*^1^Department of Structural Biology*

*Max-Planck Institute of Biophysics*

*Max-von-Laue-Str. 3*

*60438 Frankfurt am Main, Germany*

*^2^Department of Pharmacology and Systems Therapeutics*

*Mount Sinai School of Medicine*

*1 Gustave L. Levy Place*

*New York 10029, USA*

*^3^Cluster of Excellence Macromolecular Complexes*

*Max-Planck Institute of Biophysics*

*Max-von-Laue-Str. 3*

*60438, Frankfurt am Main, Germany*

**corresponding author:*

Thomas Meier

Phone: +49-69-63033038

Fax: +49-69-63033002

Email: thomas.meier@biophys.mpg.de

**Discussion on:**

Ion translocation through the *B. pseudofirmus* OF4 F_o_ complex

Ion translocation and torque generation for F_1_F_o_ ATP synthases is thought to happen in the F_o_ motor complex (Fig. 6) at the dynamic protein-protein interface when the c_13_ rotor passes by the a-subunit stator. During operation of the enzyme in the ATP synthesis mode, the hydronium ion/protonated water interacts with the glutamate (Glu54) on the rotating c-ring and enters into the rotor-stator interface in the *ion-locked* conformation (*state 0,* crystal structure presented in this work). Upon facing a more hydrophilic environment [1,2], possibly in the region of the conserved stator arginine (Arg172), the locked proton can be released into the cytoplasm [3]; the water, as an intrinsic part of the c-ring structure, is suggested to stay at the ion binding site. This leaves the carboxyl residue of Glu54 negatively charged (*state 1*). The described unlocking involves a conformational switch of Glu54 from the locked conformation to a more open orientation of the Glu54 carboxyl group with an orientation towards the outside of the c-ring and interacting with the positive charge of Arg172 (*state 2*). The conserved arginine on the stator subunit a plays a crucial role in the ion translocation process [4,5,6], it avoids ion leakage from the two access pathways to and from the binding site and stabilizes by charge compensation the outside orientation of Glu54 (*state 2*). A new proton is taken up from the periplasmic side and translocated to Glu54 by a pathway through the a-subunit [3,7,8]. In alkaliphilic *Bacillus* species proton capture is supported by the alkaliphile-specific a-subunit lysine (Lys180 in *B. pseudofirmus* OF4) that is required for ATP synthesis at alkaline pH [9,10]. The charged Glu54 can move into the re-protonation site (*state 3*) where it bonds in between Glu54 (Oε2) and the water oxygen (O). As the ion binding site is charge-compensated in the *ion-locked* conformation (*state 0*), it is able to re-enter into another rotation cycle along the hydrophobic lipid/c-ring interface.

**References (Text S1)**

1. Steed PR, Fillingame RH (2009) Aqueous accessibility to the transmembrane regions of subunit c of the *Escherichia coli* F_1_F_0_ ATP synthase. J Biol Chem 284: 23243-23250.

2. Lau WC, Rubinstein JL Structure of intact *Thermus thermophilus* V-ATPase by cryo-EM reveals organization of the membrane-bound V_O_ motor. Proc Natl Acad Sci USA 107: 1367-1372.

3. Junge W, Sielaff H, Engelbrecht S (2009) Torque generation and elastic power transmission in the rotary F_O_F_1_-ATPase. Nature 459: 364-370.

4. Eya S, Maeda M, Futai M (1991) Role of the carboxyl terminal region of H^+^-ATPase (F_0_F_1_) a subunit from *Escherichia coli*. Arch Biochem Biophys 284: 71-77.

5. Lightowlers RN, Howitt SM, Hatch L, Gibson F, Cox GB (1987) The proton pore in the *Escherichia coli* F_0_F_1_-ATPase: a requirement for arginine at position 210 of the a-subunit. Biochim Biophys Acta 894: 399-406.

6. Cain BD (2000) Mutagenic analysis of the F_0_ stator subunits. J Bioenerg Biomembr 32: 365-371.

7. Moore KJ, Fillingame RH (2008) Structural interactions between transmembrane helices 4 and 5 of subunit a and the subunit c ring of *Escherichia coli* ATP synthase. J Biol Chem 283: 31726-31735.

8. Steed PR, Fillingame RH (2008) Subunit a facilitates aqueous access to a membrane-embedded region of subunit c in *Escherichia coli* F_1_F_0_ ATP synthase. J Biol Chem 283: 12365-12372.

9. Wang Z, Hicks DB, Guffanti AA, Baldwin K, Krulwich TA (2004) Replacement of amino acid sequence features of *a*- and *c*-subunits of ATP synthases of alkaliphilic *Bacillus* with the *Bacillus* consensus sequence results in defective oxidative phosphorylation and non-fermentative growth at pH 10.5. J Biol Chem 279: 26546-26554.

10. McMillan DGG, Keis S, Dimroth P, Cook GM (2007) A specific adaptation in the *a* subunit of thermoalkaliphilic F_1_F_o_-ATP synthase enables ATP synthesis at high pH but not at neutral pH values. J Biol Chem 282: 17395-17404.
