# Supplementary figures and images for: Correction: A New Type of Proton Coordination in an F1Fo-ATP Synthase Rotor Ring
Source: PLoS Biol. 2010 Aug 20;8(8):10.1371/annotation/a025ce51-d0f4-41ca-b5b9-89d9c75cd26d. doi: 10.1371/annotation/a025ce51-d0f4-41ca-b5b9-89d9c75cd26d (PMC2927218; doi:10.1371/annotation/a025ce51-d0f4-41ca-b5b9-89d9c75cd26d)

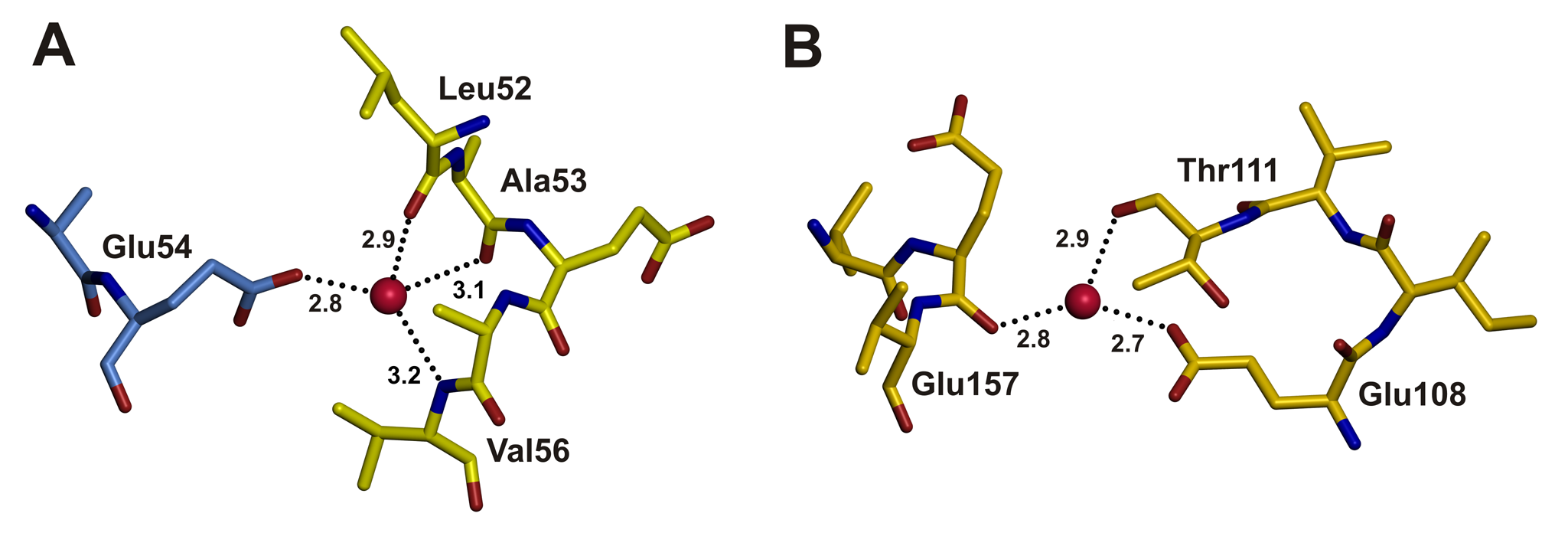

Supplement: Supplementary file 1 [file pbio.a025ce51-d0f4-41ca-b5b9-89d9c75cd26d.s001.tif]
