# Supplementary figures and images for: Correction: A New Type of Proton Coordination in an F1Fo-ATP Synthase Rotor Ring
Source: PLoS Biol. 2010 Aug 20;8(8):10.1371/annotation/4b30bafe-631f-48dd-a5c2-4e727a5853d1. doi: 10.1371/annotation/4b30bafe-631f-48dd-a5c2-4e727a5853d1 (PMC2927219; doi:10.1371/annotation/4b30bafe-631f-48dd-a5c2-4e727a5853d1)

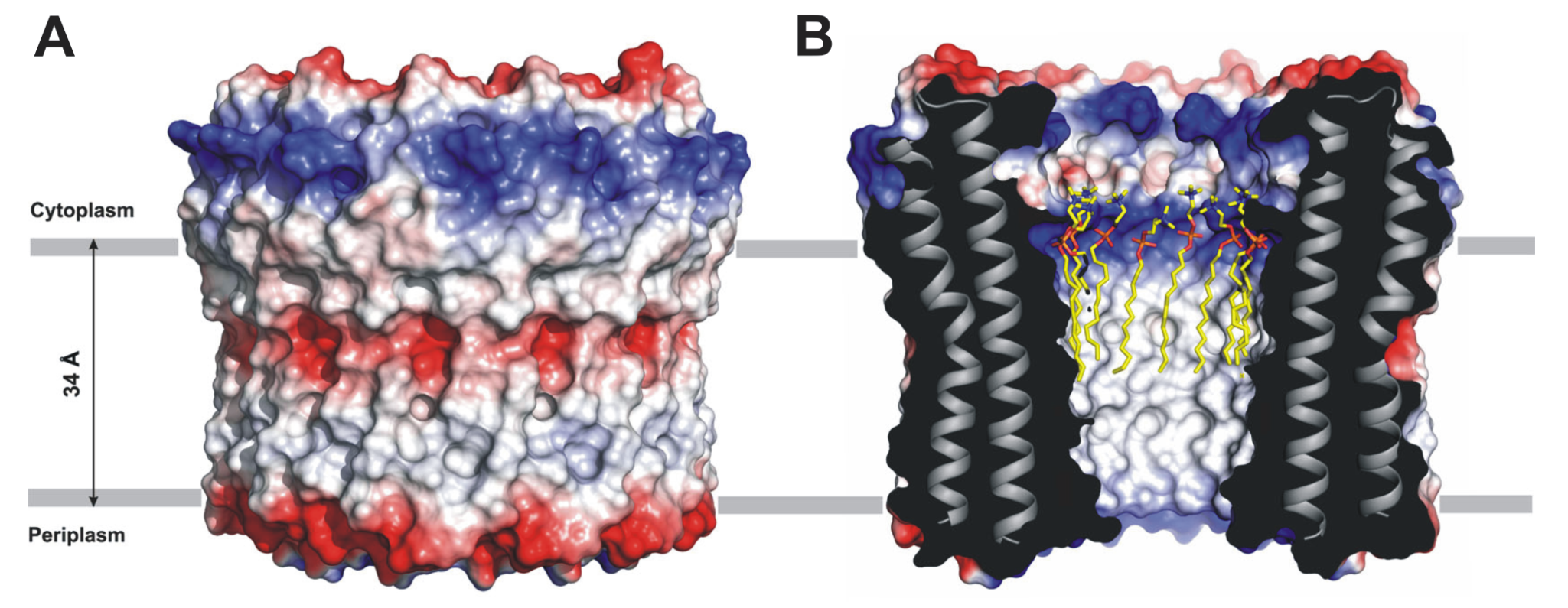

Supplement: Supplementary file 1 [file pbio.4b30bafe-631f-48dd-a5c2-4e727a5853d1.s001.tif]
